# *In vitro* characterization of the effects of chronic insulin stimulation in mouse 3T3-L1 and human SGBS adipocytes

**DOI:** 10.1080/21623945.2020.1798613

**Published:** 2020-07-27

**Authors:** A. Rossi, M. Eid, J. Dodgson, G. Davies, B. Musial, M. Wabitsch, C. Church, D.C. Hornigold

**Affiliations:** a Bioscience Metabolism, Research And Early Development, Cardiovascular, Renal and Metabolism (CVRM), BioPharmaceuticals R&D, AstraZeneca, Cambridge, UK; b Biologics Therapeutics, Antibody and Protein Engineering, R&D, AstraZeneca, Cambridge, UK; c Bioscience Renal, Research and Early Development, Cardiovascular, Renal and Metabolism (CVRM), BioPharmaceuticals R&D, AstraZeneca, Cambridge, UK; d Division of Paediatric Endocrinology and Diabetes, Department of Paediatrics and Adolescent Medicine, University Medical Center, Ulm, Germany

**Keywords:** hyperinsulinemia, 3T3-L1, SGBS, glucose uptake, lipolysis

## Abstract

Hyperinsulinemia is the hallmark of the development of insulin resistance and precedes the diagnosis of type 2 diabetes. Here we evaluated the effects of prolonged exposure (≥4 days) to high insulin doses (150 nM) *in vitro* in two adipose cell types, mouse 3T3-L1 and human SGBS. Chronic insulin treatment significantly decreased lipid droplet size, insulin signalling and insulin-stimulated glucose uptake. 3T3-L1 displayed an increased basal glucose internalization following chronic insulin treatment, which was associated with increased GLUT1 expression. In addition, both cells showed increased basal lipolysis.

In conclusion, we report the effects of prolonged hyperinsulinemia in 3T3-L1 and SGBS, highlighting similarities and discrepancies between the cell types, to be considered when using these cells to model insulin-induced insulin resistance.

## Introduction

Type 2 diabetes (T2D) is a progressive disease characterized by insulin resistance, defined as the inability of insulin to perform its functions at its target tissues, leading to an uncontrolled increase in blood glucose levels. The current available therapies for T2D are not curative but only aim at reducing hyperglycaemia. Chronically elevated levels of insulin (hyperinsulinemia) are believed to have a primary role in exacerbating and even initiating insulin resistance and the further development of frank T2D. As examples, transgenic mice carrying extra copies of insulin gene display glucose intolerance [1]. Similarly, 40 h-continuous infusion of insulin resulted in reduced glucose utilization in humans [2], and more recently hyperinsulinemia has been demonstrated to be the predominant causative factor leading to insulin resistance in type 1 diabetic subjects [3].

Adipose tissue is a key organ that responds to insulin actions and itself can contribute to aggravate insulin resistance. Insulin influences the synthesis and storage of lipids in adipocytes, free fatty acid release, glucose uptake, secretion of adipokines and cytokines. In turn, increased serum levels of fatty acids and pro-inflammatory cytokines are both associated with insulin resistance and have been demonstrated to exacerbate this condition, both *in vitro* and *in vivo* [4–6].

Primary adipocytes can be isolated or commercially obtained from both animals and humans, but adipose cell lines are cost-effective, easier to culture, without donor variation and thus are still predominantly used for basic academic research and drug discovery purposes. While 3T3-L1 preadipose cells derived from mouse embryo are the most commonly used cell line for adipose research [7], the human SGBS pre-adipocytes, originally derived from adipose tissue obtained from a patient affected by Simpson-Golabi-Behmel syndrome (SGBS) [8], have been used only in recent years. SGBS cells represent so far, the only human pre-adipose cell strain that can fully differentiate to mature adipocytes showing a comparable gene expression profile to human primary cells [8]. Recently, SGBS have been shown to differentiate towards a brown/beige phenotype offering an informative platform to study the mechanisms leading to human browning process [9].

3T3-L1 have widely been used to model *in vitro* insulin-induced insulin resistance. Various publications consistently showed that prolonged exposure to high or even physiological concentrations of insulin causes an inhibition of insulin signalling, with a consequent reduction in glucose uptake [1,3,10-12] despite no changes highlighted in either basal or insulin-mediated inhibition of lipolysis.

With this study we have characterized the *in vitro* effects of chronic hyperinsulinemia, using 3T3-L1 and the human adipose SGBS cells, which has not been evaluated before in this context. Our protocol of prolonged and repeated chronic stimulation with high insulin concentration is dissimilar from previous reports [10–14], its main purpose being to reproduce as closely as possible the hyperinsulinemia occurring *in vivo* which triggers insulin resistance.

## Materials and methods

### Cell culture and reagents

3T3-L1 pre-adipocytes (obtained from ATCC) were plated in collagen I-coated plates and grown in basal medium (DMEM with high glucose, 10% FBS and 1% penicillin/streptomycin solution) until confluence was reached. The post-mitotic cells were then differentiated into mature adipocytes using the same basal medium as specified above added with differentiation reagents as described previously [15] and kept in culture until day 11 post-induction of differentiation. Experiments were conducted on day 11.

SGBS pre-adipocytes were plated in collagen I coated plates and they were grown in DMEM/F12 medium containing 10% FBS, 1% penicillin/streptomycin solution, pantothenate (1.7 mM) and biotin (3.3 mM). Differentiation of these cells into mature adipocytes was performed using the same basal medium without FBS and added with differentiation reagents as described previously [8,16]. Experiments were conducted on day 15 post-induction of differentiation, when cells reached full adipocyte maturation.

**Figure 1.**
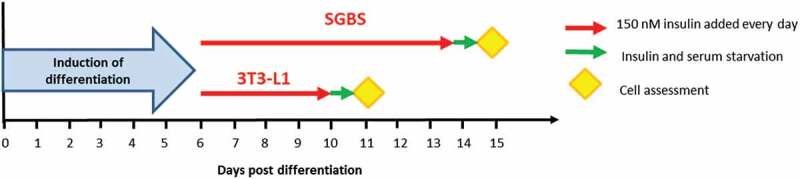
Schematic of chronic insulin stimulation protocol applied to 3T3-L1 and SGBS cells

Human recombinant insulin (Sigma Aldrich) was used for the chronic stimulation of the cells. Insulin (150 nM) was either added to fresh medium or directly spiked into wells every day from day 6 post-induction of differentiation until the day before the experiments. This concentration of insulin was chosen as it is known to elicit maximal phosphorylation of Akt in both 3T3-L1 and SGBS (Figure 4(a)). Insulin and serum starvation were performed for at least 18 h before conducting the experiments. Experiments were performed in parallel on the same day for untreated and insulin-treated cells. Detailed protocol of chronic insulin stimulation in 3T3-L1 and SGBS is shown in Figure 1.

### Cell labelling, imaging and image analysis

Cells were fixed in 3.7% paraformaldehyde at room-temperature for 30 mins followed by washing in PBS and incubation in blocking buffer (5% Donkey Serum, 0.3% Triton X-100 in PBS) at 4°C over-night. Blocking buffer was then removed and cells were incubated with either 1:200 α-Glut4 (Abcam, ab654) or 0.8 µg/ml α-Glut1 (Abcam, ab115730) primary antibodies in labelling buffer (1% BSA, 0.3% Triton X-100 in PBS) at room-temperature for 1 hour. Unbound antibody was removed by washing and cells were labelled with 1 µg/ml Hoescht 33342 (Invitrogen, 33342), 1:500 HCS LipidTox Green (ThermoFisher H34475) and 3 µg/ml α-Rabbit_AF647 (JIR, 111–605-141) secondary antibody in labelling buffer for 1 hour. Following incubation, cells were washed in PBS.

Imaging was performed on an OPERA QEHS spinning disc confocal microscope (PerkinElmer) using a X20-magnification water immersion lens (N.A. 0.7), and 405 nm, 488 nm and 640 nm lasers. Image analysis was performed using bespoke scripts created with Columbus (PerkinElmer) image analysis software. In short, lipid areas were segmented using a find image region building block. For measurement of lipid droplet size within the lipid regions individual lipid droplets were segmented and distinguished from clusters of smaller lipid droplets using a linear classifier according to the principles laid out by Dejgaard and Presley [17]. To measure intensity of GLUT4 and GLUT1 the segmented individual droplets within the lipid area were used to create an inverted mask that segmented the cytoplasmic regions of the adipocytes.

### Assessment of gene expression

Gene expression was evaluated by Real Time PCR using TaqMan gene expression assays (Thermo Fisher Scientifics) using a QuantStudio 12 K Flex Real-Time PCR System (Thermo Fisher Scientifics). Gene expression was analysed using the ∆∆Ct method using multiple housekeeping genes (PPIA, B2M, HPRT). The list of Taqman gene expression assays used in this experiment are listed in Table 1.

**Table 1.** List of human and mouse Taqman probes

Gene name	Human Taqman gene expression assay	Mouse Taqman gene expression assay
*FASN*	Hs01005622_m1	Mm00662319_m1
*SREBF1*	Hs00231674_m1	Mm00550338_m1
*LPL*	Hs00173425_m1	Mm00434764_m1
*ADIPOQ*	Hs00605917_m1	Mm00456425_m1
*LEP*	Hs00174877_m1	Mm00434759_m1
*ELOVL6*	Hs00907564_m1	Mm00851223_s1
*IL1B*	Hs01555410_m1	Mm00434228_m1
*IL6*	Hs00174131_m1	Mm00446190_m1
*PPARg*	Hs01115513_m1	Mm00440940_m1
*PPARα*	Hs00947536_m1	Mm00440939_m1
*CD36*	Hs00354519_m1	Mm00432403_m1
*CIDEA*	Hs00154455_m1	Mm00432554_m1
*CEBPα*	Hs00269972_s1	Mm00514283_s1
*PNPLA2*	Hs00386101_m1	Mm00503040_m1
*PPIA*	Hs99999904_m1	Mm02342430_g1
*B2M*	Hs99999907_m1	Mm00437762_m1
*HPRT*	Hs02800695_m1	Mm03024075_m1

### Measurement of adiponectin levels in 3T3-L1 and SGBS supernatants

Adiponectin levels were detected from the supernatants obtained from 3T3-L1 and SGBS, previously stored at −80°C, using respectively Mouse Adiponectin Kit and R-PLEX Human Adiponectin Antibody Set, both from Meso Scale Discovery, according to manufacturer’s instructions. Specifically, 3T3-L1 supernatants were diluted 1:20 and SGBS supernatants were diluted 1:100.

### Measurement of phosphorylated AKT and IRS-1 and total insulin receptor

Insulin signalling was evaluated by acute stimulation of the cells with either insulin dose response or a single dose of 100 nM insulin for 15 minutes, after which cells were lysed and protein concentration was measure with Pierce™ BCA protein assay kit (Thermo Fischer Scientifics). Normalized lysates were used to detect total and phosphorylated mouse/human Akt (Ser473 and Thr308), human total and phosphorylated IRS-1 (PY20) and human total and phosphorylated insulin receptor (PY20) using MSD® Multi-Spot Assay System (Meso Scale Discovery). Mouse total insulin receptor was detected using sandwich ELISA kit (LifeSpan Bioscience). All commercially available assays were performed according to manufacturer’s instructions.

### Measurement of glucose uptake

**Figure 2.**
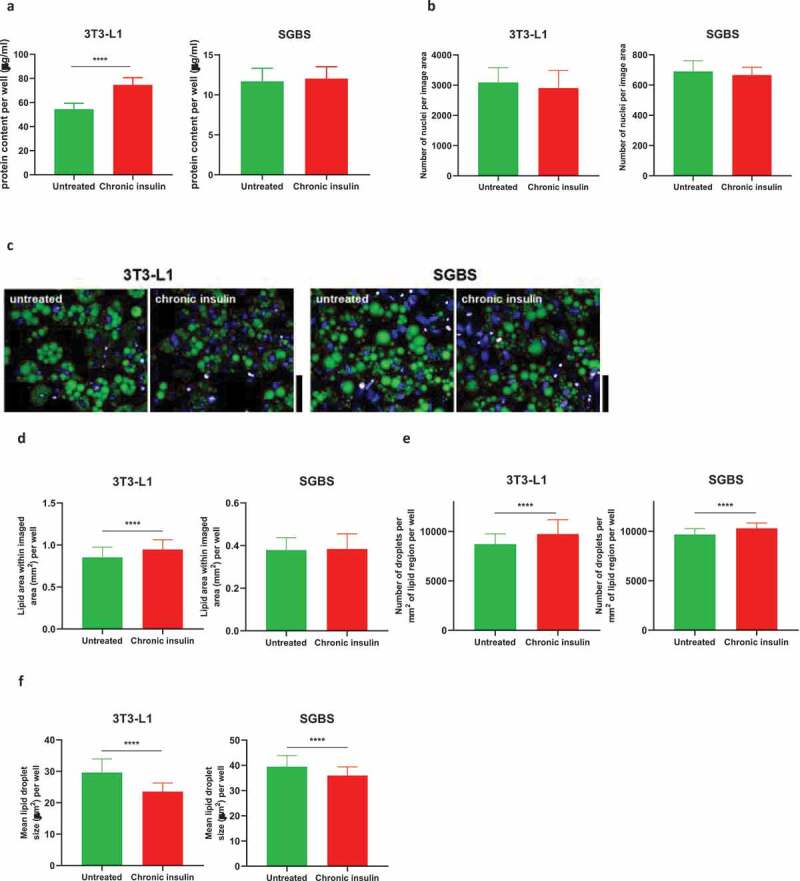
Adipocyte number and lipid accumulation following chronic insulin stimulation. (a) Total protein content per well from lysates of 3T3-L1 and SGBS obtained from 96-well plates (n ≥ 12 per condition). (b) Total nuclei number per image area in 3T3-L1 and SGBS. (c) Images of untreated and insulin-treated 3T3-L1 and SGBS mature adipocytes stained for lipid droplets with LipidTox (green) and nuclei with Hoescht (blue). Red boundary indicates areas segmented as differentiated adipocytes. Scale bar is 40 µm. (d) Area of lipid coverage within each imaging region (total area 2.2 mm^2^) per well for untreated and insulin treated 3T3-L1 and SGBS. (e) Number of lipid droplets per mm^2^ of differentiated adipocyte area per well for untreated and insulin treated 3T3-L1 and SGBS. (f) Mean size of lipid droplets for untreated and insulin treated 3T3-L1 and SGBS. Data representative of n = 3 experiments. **** indicates p < 0.001

Glucose uptake was evaluated in 3T3-L1 and SGBS cells cultured in 96-well plates using the luminescent Glucose Uptake-Glo™ Assay (Promega). The assay was performed according to the manufacturer’s instructions. Briefly, after the serum and insulin starvation period, cells were washed three times with PBS to remove traces of glucose present in the medium, and cells were stimulated with 100 nM of insulin for 30 minutes in DMEM without glucose (Thermo Fisher Scientifics). Insulin stimulation was followed by addition of 50 µl of 2DG solution, incubated for 10 minutes. 2DG6P detection reagents were then added and lysates were incubated at room temperature for 3 hours prior to detection of luminescence. 2DG6P values were not normalized by total protein as chronic insulin stimulation was found to increase the protein content specifically in 3T3-L1 (Figure 2(a)), despite cell number was maintained the same (Figure 2(b)). Therefore, unnormalized data are shown as they are believed to give a reliable result.

### Measurement of basal lipolysis

Cells were insulin and serum starved for a period of 24 hours, after which supernatants were collected and glycerol concentration was measured using the Glycerol Assay Kit (Cell-Based) (Abcam). Glycerol standard curve was prepared in the same 3T3-L1 or SGBS media used for the serum starvation and glycerol was detected as per manufacturer’s instructions. As explained above, similarly to the glucose uptake results, protein normalization was not carried out for lipolysis experiments and unnormalized results are shown.

### Statistical analyses

Graphs and statistical analyses were performed using GraphPad Prism. All data are expressed as means ± standard deviation (S.D.). Mean data from gene expression analysis were log-transformed. Statistical significance was evaluated using either unpaired Student’s t-test or One-Way ANOVA Tukey post hoc test. Differences were considered significant when p < 0.05.

## Results

### Chronic insulin treatment does not affect gross morphology, but does decrease lipid droplet size in 3T3-L1 and SGBS

To model the effects of hyperinsulinemia *in vitro* and characterize the effects of prolonged insulin exposure in adipocytes, we incubated mouse 3T3-L1 and human SGBS to a supraphysiological dose of insulin (150 nM), which was added to the medium every day from day 6 post differentiation induction until differentiation to mature adipocytes. Insulin treatment was performed at day 6 in both cell types to avoid disturbing the initial induction of differentiation. High insulin was added to 3T3-L1 until day 10, while it was added to SGBS until day 14 as these cells take longer time to differentiate to mature adipocytes. The percentage of mature adipocytes was approximately 95–100% in 3T3-L1 and 80–85% in SGBS, without obvious differences between untreated and insulin-treated cells. A schematic of the chronic insulin exposure is shown in Figure 1.

**Figure 3.**
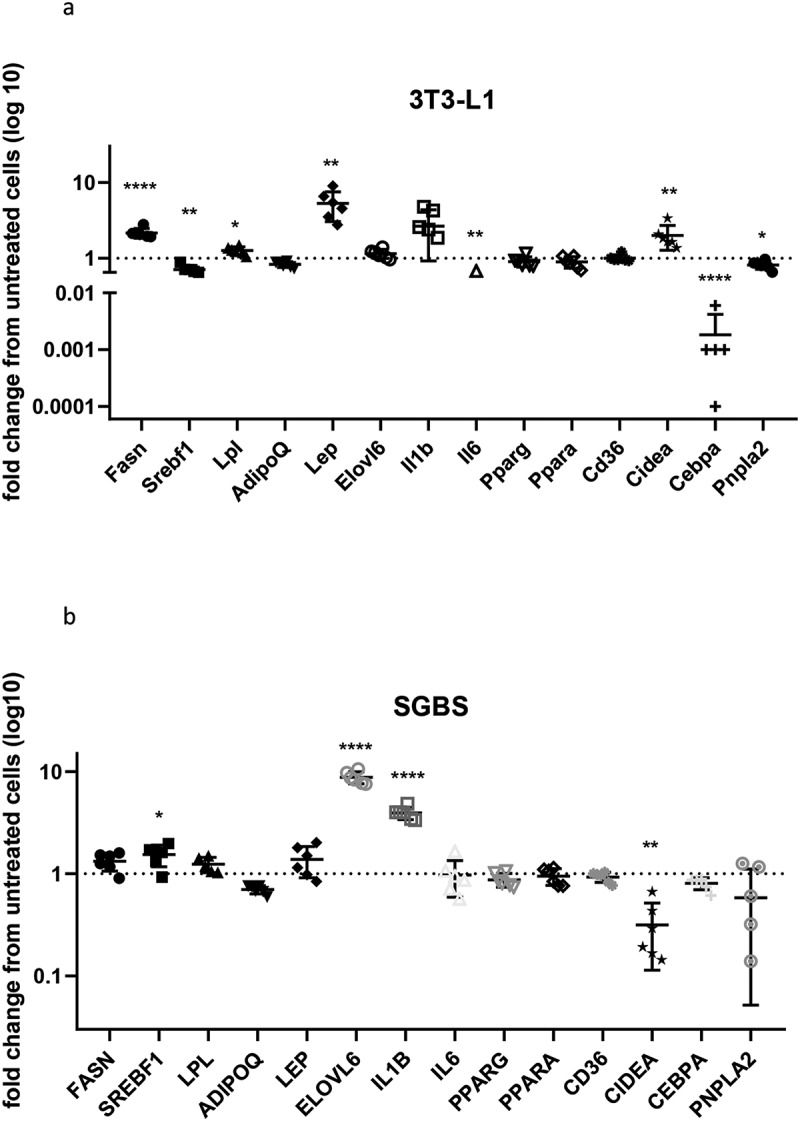
Gene expression in 3T3-L1 and SGBS. Expression of selected genes in insulin-treated 3T3-L1 (a) and SGBS (b) expressed as fold change compared to untreated cells. Data representative of n = 3 experiments. * indicates p < 0.05, ** indicates p < 0.01, **** indicates p < 0.001

The first set of experiments carried out in 3T3-L1 and SGBS chronically stimulated with high doses of insulin aimed at determining whether chronic high insulin affected total protein content, cell number and lipid accumulation. Insulin treatment caused a significant increase in total protein accumulation in 3T3-L1 but not in SGBS (Figure 2(a)). Insulin did not alter the cell number, as assessed by nuclei counts (Figure 2(b)), while lipid accumulation was marginally but significantly increased only in insulin-treated 3T3-L1 but not in SGBS (Figure 2(c,d)). PPARg gene expression was unchanged in both cell types between conditions (Table 2 and Figure 3(a,b)). These data suggest that *in vitro* hyperinsulinemia *per se* is not a sufficient stressor that can significantly affect gross cell number or differentiation but can slightly increase lipid accumulation in 3T3-L1.

Lipid droplet analysis revealed that the number of lipid droplets within the differentiated areas was significantly increased (Figure 2(e)), while the size of the lipid droplets was consistently lower in both 3T3-L1 and SGBS exposed to high insulin concentration, with the most profound effect being in 3T3-L1 (Figure 2(f)).

### Chronic insulin exposure causes distinctive gene expression changes in 3T3-L1 and SGBS

To understand the overall effects of prolonged exposure to high insulin concentrations on gene expression, a panel of 14 genes involved in various adipocyte functions (described in Table 2) was evaluated between untreated and insulin-treated cells. In general, gene expression was more affected in 3T3-L1 than in SGBS, with four genes significantly upregulated (*Fasn, Lpl, Lep, Cidea*) and four genes significantly downregulated (*Srebf1, Il6, Cebpa, Pnpla2*) by chronic insulin exposure, with the most profound effects observed for *Lep* and *Cebpa*. Distinctly, hyperinsulinemia induced an upregulation of *SREBF1, ELOVL6, IL1B* and a downregulation of *CIDEA* in SGBS (Table 2 and Figure 3).

**Table 2.** List of genes whose expression has been evaluated in 3T3-L1 and SGBS. Gene function, change in expression, fold change and p-value are reported for genes expressed in cells treated with chronic insulin as compared to untreated cells

Gene	Function	Expression in insulin- treated 3T3-L1	Fold change	p value	Expression in insulin- treated SGBS	Fold change	p value
*FASN*	lipogenesis	increased	2.16	p < 0.0001	unchanged	1.32	p = 0.07
*SREBF1*	lipogenesis	decreased	0.71	p = 0.0082	increased	1.54	p = 0.014
*LPL*	lipolysis	increased	1.26	p = 0.010	unchanged	1.24	p = 0.082
*ADIPOQ*	adipokine	unchanged	0.89	p = 0.084	unchanged	0.97	p = 0.831
*LEP*	adipokine	increased	4.2	p = 0.002	unchanged	0.691	p = 0.131
*ELOVL6*	lipogenesis	unchanged	1.15	p = 0.192	increased	8.88	p < 0.0001
*IL1B*	inflammation	unchanged	2.64	p = 0.090	increased	3.92	p < 0.0001
*IL6*	inflammation	decreased	0.58	p = 0.002	unchanged	0.97	p = 0.89
*PPARg*	adipogenesis	unchanged	0.97	p = 0.69	unchanged	0.92	p = 0.39
*PPARα*	fatty acid oxidation	unchanged	0.91	p = 0.53	unchanged	0.97	p = 0.67
*CD36*	lipid uptake	unchanged	0.99	p = 0.88	unchanged	1.07	p = 0.41
*CIDEA*	thermogenesis/lipolysis	increased	2.01	p = 0.071	decreased	0.32	p = 0.07
*CEBPα*	adipogenesis	decreased	0.0096	p = 0.0006	unchanged	0.8	p = 0.15
*PNPLA2*	lipolysis	decreased	0.83	p = 0.03	unchanged	0.58	p = 0.097

As adiponectin is the most abundant insulin sensitizing adipokine produced predominantly by adipocytes [18], we also evaluated its secretion in the supernatants obtained from 3T3-L1 and SGBS. In agreement with its gene expression profile, adiponectin secreted levels were not affected by hyperinsulinemia in both the cell types (198 ng/ml in untreated vs 199 ng/ml in insulin-treated 3T3-L1, p = 0.911; 113 ng/ml in untreated vs 95 ng/ml insulin-treated SGBS, p = 0.086).

### *Chronic insulin exposure impairs insulin signalling in both 3T3-L1 and SGBS and lead to* in vitro *insulin resistance*

**Figure 4.**
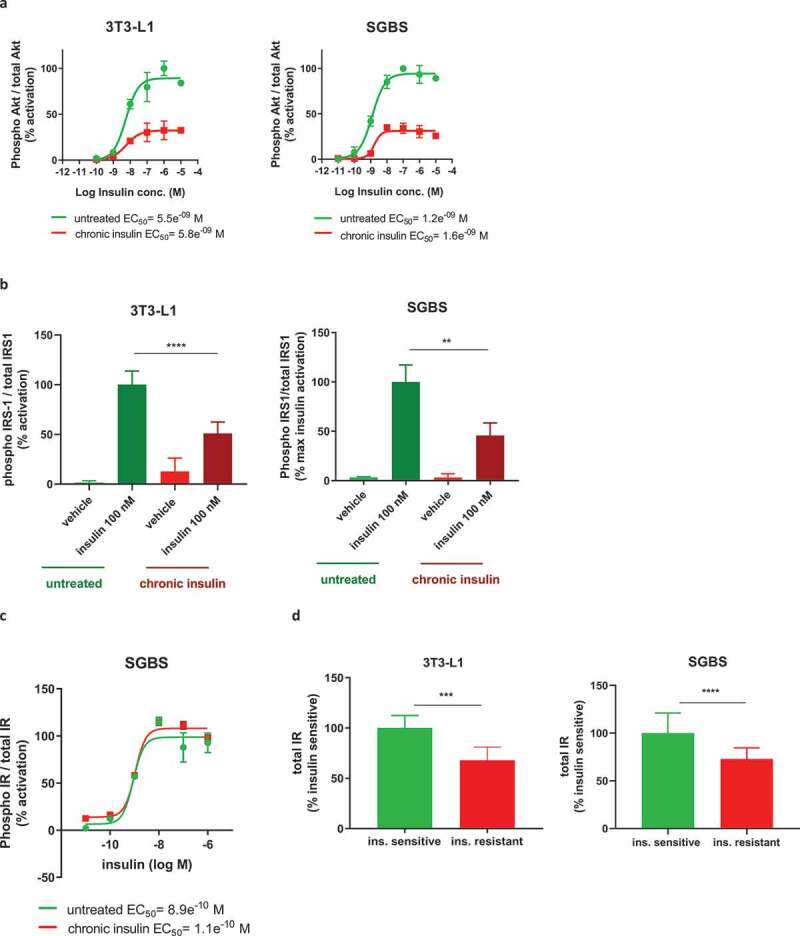
Chronic insulin treatment impairs insulin signalling in both 3T3-L1 and SGBS, causing insulin resistance. (a) Cells chronically treated with insulin and subjected to acute stimulation with insulin dose response curve show a reduction in the maximal phosphorylation of Akt but no change in insulin EC_50_. (b) Phosphorylation of IRS-1 after acute stimulation with 100 nM insulin is reduced in cells chronically treated with insulin. (c) Phosphorylation of IR is unchanged in insulin resistant SGBS subjected to acute stimulation with insulin dose response curve. (d) Protein levels of IR are decreased in both insulin resistant 3T3-L1 and SGBS. Data representative of n = 3 experiments. ** indicates p < 0.01, *** indicates p < 0.005, **** indicates p < 0.001

Hyperinsulinemia is one of the main conditions leading to the development of insulin resistance and progression to T2D *in vivo*. Thus, we asked whether our protocol of chronic insulin exposure could have induced insulin resistance by evaluating the insulin signalling pathway after acute insulin stimulation. As shown in Figure 4(a), both SGBS and 3T3-L1 previously exposed to high insulin concentration displayed a maximal phosphorylation of Akt (Ser473) which was decreased by more than 60% as compared to control cells (32.5% max activation in 3T3-L1 and 35% in SGBS), with no differences in insulin potency. Similarly, SGBS also showed reduced levels of phosphorylation of Akt at the Thr308 residue (data not shown), while this was not assessed in 3T3-L1. These data clearly demonstrate an impairment of the insulin signalling pathway, which was further corroborated by the decreased phosphorylation of the Akt upstream factor IRS-1 (determined after stimulation with a single dose of insulin, Figure 4(b)). As both 3T3-L1 and SGBS showed impaired insulin signalling following chronic exposure of high insulin concentration, such cells will be referred as insulin resistant from this point onwards.

We also measured the autophosphorylation of insulin receptor (IR) in SGBS and, in contrast to the downstream factors IRS-1 and Akt, neither its maximal phosphorylated levels nor insulin potency were affected in the insulin resistant cells (Figure 4(c)). We then assessed the total levels of IR in both 3T3-L1 and SGBS, and in this case, we found a significant decrease in both the insulin resistant cell types (Figure 4(d)). Therefore, we confirmed that hyperinsulinemia induced insulin resistance *in vitro* in both 3T3-L1 and SGBS, and it was associated with decreased levels of IR, explaining at least in part the cause of the impairment of downstream insulin signalling.

### Basal and insulin-stimulated glucose uptake is profoundly affected in insulin resistant 3T3-L1

Adipocytes account for at least 10–15% of the disposal of circulating glucose after a meal [19]. As glucose uptake is regulated by insulin-induced phosphorylated Akt [20,21], we hypothesized that insulin resistant 3T3-L1 and SGBS would show decreased internalization of glucose, as previously observed in similar models of insulin-induced insulin resistance in 3T3-L1 [10,13]. Thus, we assessed glucose uptake with a non-radioactive method using a high-sensitive ultra-luminescence kit.

**Figure 5.**
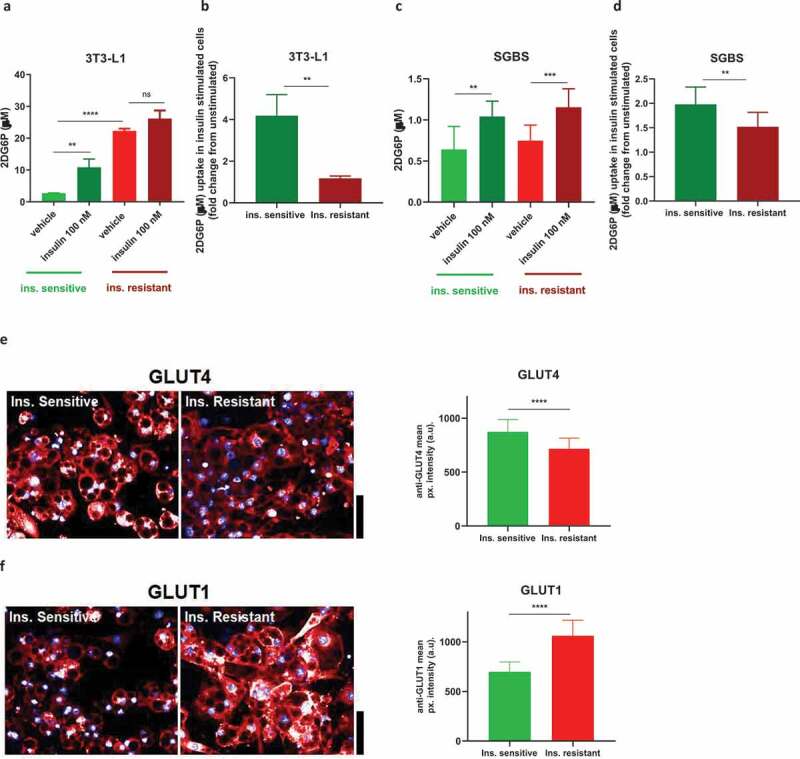
Insulin resistance affects glucose uptake in 3T3-L1 and SGBS. Insulin resistant 3T3-L1 show increased basal and reduced insulin-stimulated glucose uptake (a,b) while SGBS show only minor impairment in insulin-stimulated glucose uptake (c,d). (e) Representative images and graphs quantifying GLUT4 levels in unstimulated insulin sensitive and resistant 3T3-L1. (f) Representative images and graphs quantifying GLUT1 levels in unstimulated insulin sensitive and resistant 3T3-L1. All scale bars are 40 µm. Data representative of n = 3 experiments. ** indicates p < 0.01, *** indicates p < 0.005, **** indicates p < 0.001

As shown in Figure 5(a), the insulin sensitive 3T3-L1 acutely stimulated with 100 nM insulin were characterized by a fourfold increase in 2DG6P uptake. The insulin resistant 3T3-L1 displayed an increase of approximately 10-fold in the basal uptake of glucose compared to the insulin sensitive cells, with only a modest increase in insulin-stimulated glucose uptake. In Figure 5(b) a direct comparison of the 2DG6P uptake induced by insulin expressed as fold change reveals also an impaired insulin-stimulated glucose uptake in the insulin resistant cells. In insulin resistant SGBS the basal glucose uptake was not different compared to insulin sensitive cells, while a small but significant decrease in insulin-stimulated glucose uptake (when expressed as fold change) was observed in the insulin resistant cells (Figure 5(c,d)).

To understand the possible reasons underlying these differences in glucose uptake in the insulin resistant 3T3-L1, we looked at the protein expression of the two main adipocyte glucose transporters, GLUT4 and GLUT1 by immunofluorescence. GLUT4 is the main glucose transporter that translocates from the cytoplasm to the cell membrane upon insulin stimulation and Akt phosphorylation, mediating glucose access into the cell, while GLUT1 is another transporter abundantly expressed in 3T3-L1, localized both in the cytoplasm and cell membrane and its translocation to the cell surface has been found to be minimal in response to insulin [14].

As shown in Figure 5(e), imaging showed a reduction in total levels of GLUT4 in insulin resistant 3T3-L1 (fold change 0.82), with GLUT4 mainly localized in the cytoplasm as expected in unstimulated cells. Most importantly, GLUT1 levels were significantly increased in insulin resistant 3T3-L1 (fold change 1.52) with clear localization to the cell membrane (Figure 5(f)). In SGBS, expression of both GLUT4 and GLUT1 were not significantly affected (data not shown), in line with the minimal effects seen in glucose uptake in this cell strain.

### Basal lipolysis is increased in insulin resistant 3T3-L1 and SGBS

**Figure 6.**
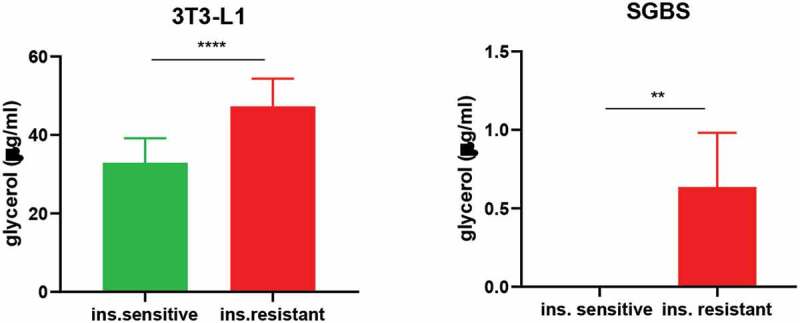
Basal lipolysis is increased in insulin resistant 3T3-L1 and SGBS. Data representative of n = 3 experiments. ** indicates p < 0.01, *** indicates p < 0.005, **** indicates p < 0.001

Increased adipose tissue lipolysis is a critical function of insulin resistance, as it leads to ectopic fat accumulation and further impairs insulin signalling in peripheral tissues [22,23]. Along with glucose uptake, we also determined whether our protocol of insulin-induced insulin resistance could affect this cellular function. Glycerol released in the supernatant of unstimulated 3T3-L1 and SGBS was evaluated after 24 hours of serum and insulin starvation. As shown in Figure 6, both insulin resistant cells showed elevated free glycerol measured in the supernatant compared to the insulin sensitive condition. The data also highlight a difference in the basal lipolysis between the two cell types, with insulin sensitive SGBS displaying no detectable glycerol release. Overall, these data suggest that *in vitro* hyperinsulinemia and the associated insulin resistance increase basal lipolysis of adipocytes, reflecting well the *in vivo* situation.

## Discussion

Multiple lines of evidence have highlighted how hyperinsulinemia is a pathophysiological condition that, if not reversed, leads to insulin resistance which, subsequently, can progress to frank T2D [1].

Several studies have explored *in vitro* models of insulin resistance using stressors of different nature (pro-inflammatory agents, lipids, cytokines, hormones) and discerned their distinct roles in a range of cell types, including adipocytes [1,3,10,12,24-26]. However, the studies evaluating the specific effects of high insulin concentrations in adipose cell lines have been limited: only 3T3-L1, and not SGBS, have been assessed as adipose cellular model, and the treatment with insulin was often conducted with a low concentration of the hormone (10 nM^11,26^, a concentration that, according to our phospho-Akt experiments, is close to insulin EC_50_ thus it does not trigger maximal Akt phosphorylation), for a brief period of time (minimum 12 hours [12] maximum 24 hours [10,13,26]) and with shorter starvation period (between 2 hours [11,13]and 8 hours [26]). To our knowledge, this is the first study where a protocol of chronic (4 to 8 days) treatment with high concentration of insulin (150 nM, well above insulin EC_50_ in Akt phosphorylation assays) has been applied to the two adipose cell types mouse 3T3-L1 and human SGBS, both extensively used for basic research and drug discovery. With this aggressive protocol, we believe we have a more accurate representation of hyperinsulinemia as it occurs *in vivo*. Moreover, our prolonged starvation period allows to maximize the insulin assay window as well as detect the differences genuinely caused by previous insulin hyperstimulation, by allowing cells to come back to their basal, unstimulated status.

The results obtained indicate that hyperinsulinemia did not alter gross cell number of cells or differentiation to mature adipocytes. Insulin treated 3T3-L1 (but not SGBS) showed increased lipid accumulation, in agreement with the increased expression of FASN, enzyme that drives lipogenesis and is known to increase in response to insulin anabolic actions [27]. In addition, it was found that insulin-treated cells displayed reduced lipid droplet size, in agreement with increased basal lipolysis (discussed below).

We assessed expression of selected genes involved in adipocyte differentiation and functions. The distinct changes in gene expression between 3T3-L1 and SGBS highlight specific responses to hyperinsulinemia from the two cell types. In particular, we observe altered expression of genes previously reported to aggravate insulin resistance in both cell types, with the most profound changes seen for *Cebpa* [28] (fold change 0.0096) and *Lep* [29] (fold change 4.2) in 3T3-L1, *ELOVL6* [30] (fold change 8.88) and *IL1B* [31] (fold change 3.92) in SGBS. Upregulation of genes involved in maintaining insulin sensitivity was also revealed, with the most significant changes observed for *CIDEA* (fold change 0.32) in SGBS [32]. We hypothesize that the increased expression of this insulin sensitizing genes acts as a counter mechanism to contrast the hyperinsulinemia-induced insulin resistance. Of note, there are similarities and discrepancies in gene expression changes between our insulin resistance protocol and the published chronic insulin treatments. In particular, while *Fasn* was upregulated in 3T3-L1 in agreement with a previous report, genes coding for the insulin sensitizers adiponectin and *PPARg* were unchanged in our insulin resistant 3T3-L1 [10]. Surprisingly, adiponectin was found to be unaffected in both 3T3-L1 and SGBS supernatants, suggesting that insulin resistance caused by chronic insulin stimulation is not due to altered expression or secretion of this crucial insulin sensitizing adipokine.

Importantly, in line with previous studies [10–13,26], our protocol successfully induced insulin resistance in both adipose cell types as demonstrated by the 50% decrease in insulin-mediated phosphorylation of Akt and IRS-1, without changes in insulin potency. While IR phosphorylation was the same between insulin sensitive and insulin resistant SGBS, we observed a reduction of the total expression of IR, which could be at least in part responsible for the impaired downstream insulin signalling and it was not highlighted in previous models of insulin-induced insulin resistance, despite two studies reported a decrease of insulin binding to its receptor after inducing hyperinsulinemia *in vitro* [33,34]. Similar to our results, Branmark et al. compared insulin signalling in human adipocytes isolated from healthy and T2D subjects and the results revealed no differences in IR phosphorylation (same insulin EC_50_) but impaired downstream insulin signalling [35].

Further novel results come from our functional assay studies. As previously shown [11,12], prolonged treatment with high insulin concentration decreased insulin-stimulated glucose uptake and GLUT4 protein levels in 3T3-L1. Somewhat surprising was the increase in the basal glucose uptake in insulin resistant 3T3-L1, which was not observed in any previous publications [10–13] and seems to be specific of our protocol. As we observed a substantial increase in the protein level of GLUT1 by immunofluorescence, and we noticed that it was mostly localized at the membrane without need of insulin stimulation, we hypothesize that this is the transporter responsible for the increased basal glucose internalization, although we acknowledge that other techniques, such as western blot, would have been more sensitive to assess the difference in GLUT1 protein expression. Interestingly, ERK1/2 activation has been shown to contribute to increase GLUT1 levels in 3T3-L1 [36]. Insulin resistant SGBS show only moderate changes in glucose uptake, highlighting once again how the different adipocyte background can influence the response to hyperinsulinemia. This finding was somewhat unexpected given the decrease in insulin-mediated Akt phosphorylation, but it was in agreement with unchanged expression of GLUT4 and GLUT1. It is possible that SGBS need a longer exposure to high insulin stimulation to see more pronounced effects of hyperinsulinemia on glucose uptake. It has also to be highlighted that SGBS display a general lower basal and insulin-stimulated 2DG6P internalization compared to 3T3-L1.

In addition, we found higher basal lipolysis in insulin resistant 3T3-L1 and SGBS. This represents a novel finding, as both basal and insulin-stimulated lipolysis were previously reported to be unchanged [11] and highlights how essential the treatment with insulin is in a prolonged and persistent manner to maximize the effects of hyperinsulinemia. This result correlates well with the decreased size in lipid droplets quantified from imaging. This *in vitro* result also mirrors insulin resistance as it occurs *in vivo* as insulin resistance in humans is also associated with higher circulating free fatty acids that can promote ectopic fat accumulation hence further worsening insulin sensitivity at the whole-body level [22,23,37].

It is also important to notice that in our protocol the cellular consequences of chronic insulin treatment at the signalling and functional levels were all detected after a period of at least 18 hours of serum deprivation in 3T3-L1 cells and insulin starvation in both cell types, suggesting that the length of the starvation period might be another variable modulating the detrimental effects of hyperinsulinemia published here and elsewhere [10–13,26].

In general, our data show that hyperinsulinemia does not necessarily cause the same cellular response between 3T3-L1 and SGBS, as demonstrated by the divergent gene expression and glucose uptake results. We hypothesize that these differences may be due to the distinct cell background, culture condition, days of insulin stimulation and species. Potentially, an even longer insulin treatment could level such discrepancies and lead to the same outputs in the two cell models, as well as give a closer representation of insulin resistance *in vivo*. Unfortunately, mature 3T3-L1 and SGBS, similarly to primary adipocytes, have a limited lifespan in culture and such experiment is currently not possible.

The main limitation of this study is the lack of comparison with primary mouse and human adipocytes, to understand whether 3T3-L1 and SGBS have the same response of primary cells subjected to hyperinsulinemia thus representing reliable cellular models.

The 3T3-L1 and SGBS *in vitro* models of insulin-induced insulin resistance have been generated with the specific aim to mirror, as closely as possible, the condition of prolonged hyperinsulinemia on adipocytes as it occurs *in vivo*, and potentially to support clinical significance by understanding mechanism of therapeutic candidates in this hyperinsulinemia setting. While the results obtained confirm that chronically elevated insulin levels are sufficient to have a detrimental effect on adipocyte insulin sensitivity by negatively affecting glucose internalization and increasing basal lipolysis locally within the adipose tissue, they could also lead to metabolic consequences at the whole body level, such as hyperglycaemia and ectopic fat accumulation, which in turn would aggravate insulin resistance in various tissues and facilitate the progression to frank T2D. However, further experiments where our protocol of chronic insulin stimulation is complemented with other factors known to exacerbate insulin resistance in T2D patients, such as fatty acids, corticosteroids and pro-inflammatory cytokines, would be required to potentially further enhance an established condition of insulin resistance as it physiologically happens in humans.

In conclusion, we have reported a novel and more aggressive insulin-induced insulin resistance protocol applied to mouse 3T3-L1 and human SGBS, characterizing their specific cellular, molecular and functional responses which need to be considered when choosing the cell model to use, and highlighting similarities and discrepancies with other previously published protocols.
